# Association of loneliness and perceived social exclusion with donation behavior among community-dwelling individuals aged 40 and over: longitudinal evidence from the nationally representative German Ageing Survey

**DOI:** 10.1007/s40520-026-03321-7

**Published:** 2026-01-25

**Authors:** André Hajek, Dong Keon Yon, Pinar Soysal, Karl Peltzer, Supa Pengpid, Hans-Helmut König

**Affiliations:** 1 Department of Health Economics and Health Services Research, University Medical Center Hamburg-Eppendorf, Hamburg Center for Health Economics, Hamburg, Germany; 2 Center for Digital Health, Medical Science Research Institute, Kyung Hee University College of Medicine, Seoul, South Korea; 3 Department of Geriatric Medicine, Faculty of Medicine, Bezmialem Vakif University, Istanbul, Türkiye; 4 Department of Health Education and Behavioral Sciences, Faculty of Public Health, Mahidol University, Bangkok, Thailand; 5 Department of Psychology, University of the Free State, Bloemfontein, South Africa; 6 Department of Medical Research, China Medical University Hospital, China Medical University, Taichung, Taiwan; 7 Department of Public Health, Sefako Makgatho Health Sciences University, Pretoria, South Africa; 8 Department of Healthcare Administration, College of Medical and Health Science, Asia University, Taichung, Taiwan

**Keywords:** Loneliness, Social isolation, Social exclusion, Donation, Prosocial behavior, Generous activities, Altruism, Germany, Mental health, Depression

## Abstract

**Background:**

There is limited knowledge regarding the association of loneliness or social exclusion with donation behavior.

**Aims:**

Our aim was to investigate the association of loneliness and perceived social exclusion with donation behavior based on a longitudinal approach.

**Methods:**

Longitudinal data were taken from wave 5 (year 2014) to wave 8 (year 2023) of the German Ageing Survey encompassing community-dwelling individuals aged 40 years and over. Established tools were used to quantify loneliness and perceived social exclusion. The willingness to donate and the total amount (in euros) of all donations (in the last 12 months) served as outcome measures. Fixed effects (FE) regressions with cluster-robust standard errors were used.

**Results:**

FE regressions showed that increases in loneliness were significantly associated with lower odds of donation after adjusting for socioeconomic factors, but became insignificant when accounting for lifestyle and health-related covariates. Conversely, a significant association was found between increases in perceived social exclusion and lower odds of donation across all models. However, neither changes in loneliness nor perceived social exclusion were significantly associated with changes in log amount of donations. Sociodemographic factors (i.e., age, sex, and education) did not moderate the identified associations.

**Discussion:**

Even after adjusting for a wide array of covariates, our longitudinal study showed a significant and robust association between increases in social exclusion and lower odds of donation.

**Conclusions:**

Avoiding increases in perceived social exclusion could encourage the decision to donate, an important prosocial behavior, pending further longitudinal evidence. Cross-country comparisons are recommended.

## Introduction

There is an ongoing demographic ageing in various countries globally. In later life, several life events can take place such as losing friends and relatives, health deteriorations, loss of autonomy or nursing home admissions – which can ultimately result in loneliness [1–5]. Loneliness refers to a perceived discrepancy between actual and desired social relationships [6], whereas perceived social exclusion refers to a feeling of being excluded from society [7].

Previous studies have already identified potential consequences of loneliness and social exclusion such as worsening sleep quality [8], lower expected longevity [9], multimorbidity [10] or mortality [11]. In addition to such aforementioned health outcomes, loneliness and exclusion can also have an impact on our prosocial behavior (i.e., any action that benefits others such as helping, sharing, cooperating or volunteering [12]). Donation behavior is also a specific form or an example of prosocial behavior that can be driven by both altruistic and other prosocial motives. For example, individuals can donate to charities, social organisations, or humanitarian groups and thus directly help vulnerable groups. Donations also refer to actual behaviour and not just prosocial thoughts. In other words: Donations directly show care and empathy for those in need. Furthermore, they can contribute to overcoming common challenges (such as natural disasters) and can promote social cohesion.

In Germany, approximately 22.7 million people aged 50 to 90 made donations in 2020/21 [13]. The total value of these donations was around 9.2 billion euros in 2021 [13]. Furthermore, around 90% of donations were between 50 and 1,000 euros [13]. It has been shown that the odds of donating were positively associated with, among other things, being male, having a higher income and having higher education in Germany [13].

Generally, there are several studies examining the association of loneliness or social exclusion with prosocial behavior (in a broad sense). For example, a recent meta-analysis revealed a weak negative correlation between social exclusion and prosocial behavior (*r*=-0.10, 95% CI: − 0.17 to − 0.04) [14]. A similar negative correlation (*r*=-0.12, 95% CI: − 0.19 to − 0.05) was identified between loneliness and prosociality in another meta-analysis [15]. However, much less is known about the association of loneliness or social exclusion with *donation behavior*, more specifically. For example, in an experimental study based on students aged 19 to 33 years in Seoul (South Korea), Lee and Park showed that individuals experiencing social exclusion had lower donation intentions and donated less [16]). Other experimental studies based on students showed similar findings ( [17, 18]. Another cross-sectional observational study found an association between higher social exclusion levels and lower donation intentions based on a selective sample of 216 individuals aged 40 years and over living in South Korea [19]. In sum, there are only a few experimental studies (with limitations regarding external validity in terms of population validity and ecological validity) and almost no observational studies (particularly based on longitudinal and nationally representative data) investigating the association of loneliness or social exclusion with donation behavior. Therefore, we aimed to examine the association of loneliness, and perceived social exclusion with donation behavior based on longitudinal data from a nationally representative sample of community-dwelling individuals aged 40 years and over. Our findings may make an important contribution to a better understanding of the association between loneliness and exclusion and donation behavior, an important prosocial behavior. Efforts to increase donations are of importance because they may help those in need and may promote social justice. Furthermore, they may improve cohesion within communities. Furthermore, they may support charitable projects and may enable innovation.

Potential explanations for an association of loneliness and perceived social exclusion with unfavorable donation behavior refer to the potential cognitive distortions that may accompany loneliness and exclusion [20]. For example, lonely and excluded people tend to be more sceptical of state institutions [21, 22]. They are also more likely to believe in conspiracy theories [23] and may also be more distrustful of, for example, charitable organizations. Moreover, because socially excluded individuals in particular may feel that they are not treated well by society in general, it may be the case that they would not want to give anything back to society (in the sense of donations, according to the reciprocity theory). Society and the individuals living in it may be somewhat less important to them. For example, such individuals might be somewhat less inclined to think about others in the event of natural disasters. They may also have fewer social occasions or community experiences, which may decrease the likelihood of donating. However, there are also arguments for an association of loneliness and perceived social exclusion with favorable donation behavior. Lonely and excluded individuals may donate to regain social recognition or a sense of belonging. For example, such individuals might see donations as an act of reintegration into society. They may also strive to prevent others from suffering the same fate (in terms of loneliness and social exclusion) as they did, which is why they may donate. Overall, however, we assume that loneliness and exclusion may contribute to unfavorable donation behavior.

## Methods

### Sample

Our present study used data from wave 5 (2014), wave 6 (2017), wave 7 (2020/2021), and wave 8 (wave 8, data collection from December 2022 to June 2023) of the very renowned German Ageing Survey (DEAS). This nationally representative study encompasses community-dwelling individuals aged 40 years and above in Germany. Thus, the main inclusion criteria are that individuals are 40 years and older and are residing in private households (implying that individuals residing in institutionalized settings were excluded). Following individuals over time (i.e., panel participants) required one or more valid interviews in previous waves, willingness to participate, being alive and residing in Germany. In our study, we focused on these panel participants (from wave 5 to 8), i.e., without further restrictions.

Several topics related to aging are included in the DEAS study such as ageism, health, family, housing, digitalization, or retirement. For example, the response rate was about 62% in the most recent wave 8.

For instance, average interviews (including general topics such as sociodemographics) took about 81 min in wave 8. An additional questionnaire (mostly including more sensitive questions) could be completed (online or by mail) in each wave. Approximately 80 to 85% usually completed the drop-off (e.g., in wave 8: 84.4%). Further details are presented in Klaus et al. [24].

All individuals included in the DEAS study provided informed consent. This study is in accordance with the Declaration of Helsinki and its later amendments. It is worth noting that an ethical review for the DEAS study was not needed because the criteria for the need of such a vote was not met. For example, the study did not involve patient examinations or the use of invasive methods.

### Outcomes: donation behavior

Individuals were asked about their donation behavior. More precisely, the wording was as follows: “Some people make occasional or regular donations to charitable, social, or humanitarian causes. Please think about it: Have you made any donations in the past 12 months?” (yes or no). Individuals responding with “yes” were asked “How high was the total amount of these donations in the past 12 months?” (approx. _ _ _ _ _ _ _ _ _ _ Euro). Amounts of money starting at 1 euro were allowed. We calculated the natural logarithm of it due to its skewness. This log total amount of donations served as second outcome. It is worth noting that the aforementioned questions used to quantify donation behavior are similar to items used in other large cohort studies [25].

### Independent variables of interest: loneliness and social exclusion

In this study, loneliness was assessed using the well-established De Jong Gierveld Loneliness Scale [26], encompassing six items. The overall loneliness score was calculated by averaging responses to all six items. This score ranges from 1 to 4 (higher scores denote greater loneliness). The scale demonstrated good internal consistency (e.g., in the most recent wave, Cronbach’s alpha was 0.84).

To measure perceived social exclusion, a four-item instrument developed by Bude and Lantermann [7] was employed. Exemplary items are: “I feel excluded from society” or “I feel like I do not really belong to the society”. The perceived social exclusion score was obtained by averaging responses across the four items, with scores varying from 1 to 4 (higher scores correspond to higher perceived social exclusion). This tool also showed strong internal consistency (e.g., Cronbach’s alpha equalled 0.87 in the most recent wave). The Pearson correlation between the outcomes is *r* = 0.52 in our present study.

### Covariates

Based on previous research [27–29], the following time-varying (i.e., factors that vary within individuals included in our sample over time) socioeconomic covariates were included in fixed effects (FE) regression analysis: age, marital status (married and living together with spouse, married but living separated from spouse, single, widowed, or divorced), employment status (employed, retired, other), and household net equivalent income (in euros). With regard to time-varying lifestyle-related covariates, frequency of sports activity, alcohol intake (in both cases: daily; several times a week; once a week; 1–3 times a month; less often; never), and smoking status (yes, daily; yes, sometimes; no, not anymore, no, never) were included in FE regression analysis. With regard to time-varying health-related covariates, self-rated health, physical functioning, depressive symptoms and the number of chronic conditions were included in FE regression analysis. Self-rated health was assessed using an established single-item tool (varying from 1 = very good to 5 = very poor). Physical functioning was measured using the SF-36 subscale physical functioning (consisting of ten items) [30]. The score varies from 0 to 100 (whereby higher values reflect better physical functioning). A count score of eleven chronic conditions (cardiac and circulatory disorders; bad circulation; joint, bone, spinal or back problems; respiratory problems, asthma, shortness of breath; stomach and intestinal problems; cancer; diabetes; gall bladder, liver or kidney problems; bladder problems; eye problems, vision impairment; ear problems, hearing problems) corresponds to the presence or absence of those conditions.

The time-constant factors (i.e., they do not vary within individuals included in this sample over time) sex (men, women) and education (according to the International Standard Classification of Education (ISCED)-classification [31]: three categories: low, medium or high) were used for descriptive purposes and as moderating factors in FE regression analysis.

### Statistical analysis

Sample characteristics are first depicted. Then, unadjusted and adjusted conditional FE logistic (with the dichotomous outcome donation; yes or no) regressions among the total sample were first estimated. Moreover, unadjusted and adjusted log-linear FE regressions were then estimated (with the continuous outcome log total amount of donations) among individuals donating (i.e., the donors). The first adjusted model incorporated socioeconomic covariates, followed by a model introducing lifestyle-related covariates. Finally, health-related covariates were also integrated in the FE regression model.

Compared to other popular panel regression models such as random effects (RE) regressions, FE regressions offer the advantage of providing consistent estimates even when time-constant factors (unobserved and observed) are systematically associated with the regressors. Our choice was also substantiated by Hausman-tests. A key characteristic of FE regression is that they solely rely on changes within individuals over time (e.g., loneliness changes within individuals over time or changes in donation behavior within individuals over time). Hence, only time-varying factors such as loneliness can be included as main effects in FE regressions. However, it was tested whether key sociodemographic factors (sex, education, and age) moderate the association of loneliness and perceived social exclusion with donation behavior by including respective interaction terms. The proportion of missings was very low (e.g., 0.8% in loneliness, 0.7% in perceived social exclusion, or 0.4% in donation in wave 8).

Statistical significance was determined with a p-value less than 0.05. StataNow 19.5 MP-Parallel Edition (StataCorp, College Station, Texas) was used for all statistical analyses.

## Results

### Sample characteristics

Sample characteristics (pooled analytic sample, *n* = 5,716 observations) are presented in Table 1. The mean age was 66.5 years (SD: 10.6 years, varying from 40 to 99 years), with 49.5% being female. Mean loneliness score was 1.8 (SD: 0.6), and mean perceived social exclusion score was 1.6 (SD: 0.6). In total, 52.5% donated. Thereof, the mean total amount of donations equaled 240.9 Euro (SD: 829.4 Euro). Additional details are shown in Table 1.

**Table 1:** Sample characteristics (pooled analytic sample = 5,716 observations)

Variables	Mean (SD) / N (%*)*
Age: Mean (SD)	66.5 (10.6)
Sex: N (%)	
Men	2888 (50.5)
Women	2828 (49.5)
Education (ISCED-classification): N (%)	
Low	255 (4.5)
Medium	2910 (50.9)
High	2548 (44.6)
Marital status: N (%)	
Married, living together with spouse	3947 (69.1)
Married, living separated from spouse	86 (1.5)
Divorced	602 (10.5)
Widowed	735 (12.9)
Single	346 (6.1)
Employment status: N (%)	
Employed	1954 (34.2)
Retired	3422 (59.9)
Other: not employed	340 (5.9)
Household net equivalent income (in Euro): Mean (SD)	2094.1 (1348.0)
Frequency of sports activity: N (%)	
Daily	494 (8.6)
Several times a week	1611 (28.2)
Once a week	991 (17.3)
1–3 times a month	373 (6.5)
Less often	671 (11.7)
Never	1576 (27.6)
Alcohol intake: N (%)	
Daily	615 (10.8)
Several times a week	1416 (24.8)
Once a week	923 (16.1)
1–3 times a month	702 (12.3)
Less often	1431 (25.0)
Never	629 (11.0)
Smoking status: N (%)	
Yes, daily	719 (12.6)
Yes, sometimes	224 (3.9)
No, not anymore	2245 (39.3)
No, never	2528 (44.2)
Self-rated health (from 1 = very good to 5 = very poor): Mean (SD)	2.5 (0.8)
Physical functioning (from 0 to 100, with higher scores reflecting better physical functioning): Mean (SD)	82.4 (22.0)
Number of chronic conditions (from 0 to 11): Mean (SD)	2.6 (2.0)
Depressive symptoms (from 0 to 45, with higher values reflecting more depressive symptoms): Mean (SD)	6.5 (6.1)
Loneliness (from 1 to 4, with higher values reflecting higher loneliness levels): Mean (SD)	1.8 (0.6)
Perceived social exclusion (from 1 to 4, with higher values reflecting higher perceived social exclusion levels): Mean (SD)	1.6 (0.6)
Donation: N (%)	
No	2702 (47.3)
Yes	3014 (52.7)
Total amount of donations (in Euro): Mean (SD)	240.9 (829.4)
(Log) total amount of donations (in Euro): Mean (SD)	4.6 (1.2)

### Regression analysis

Unadjusted and adjusted FE regressions are displayed in Table 2 (with the dichotomous outcome donation) and Table 3 (with the continuous outcome log total amount of donations).

**Table 2:** Association of loneliness, and perceived social exclusion with donation (no or yes) among the total sample. Results of conditional logistic FE regressions (DEAS, waves 5 to 8)

	Donation	Donation	Donation	Donation	Donation	Donation	Donation	Donation
Loneliness	0.83*	0.83*	0.85+	0.87				
	(0.71–0.98)	(0.70–0.98)	(0.72–1.01)	(0.73–1.04)				
Perceived social exclusion					0.86*	0.85*	0.85*	0.85*
					(0.75–0.98)	(0.73–0.97)	(0.73–0.98)	(0.73–0.99)
Socioeconomic covariates		✓	✓	✓		✓	✓	✓
Lifestyle-related covariates			✓	✓			✓	✓
Health-related covariates				✓				✓
Observations	6,414	6,002	5,899	5,716	6,440	6,027	5,918	5,723
Individuals	1,959	1,841	1,817	1,773	1,959	1,841	1,816	1,769
Pseudo R²	0.001	0.01	0.02	0.02	0.001	0.01	0.02	0.02

Comments: Unstandardized beta coefficients are shown, with 95% CI in parentheses; *** *p* < 0.001, ** *p* < 0.01, * *p* < 0.05, + *p* < 0.10

Socioeconomic time-varying covariates encompass age, marital status, employment status, and household net equivalent income; lifestyle-related time-varying covariates encompass alcohol intake, smoking status, and frequency of sports activity; health-related time-varying covariates encompass self-rated health, physical functioning, depressive symptoms and number of chronic conditions

**Table 3:** Association of loneliness, and perceived social exclusion with log total amount of donations in Euro among individuals donating. Results of log-linear FE regressions (DEAS, waves 5 to 8)

	Log total amount of donations	Log total amount of donations	Log total amount of donations	Log total amount of donations	Log total amount of donations	Log total amount of donations	Log total amount of donations	Log total amount of donations
Loneliness	0.01	0.01	0.01	0.01				
	(-0.04–0.07)	(-0.04–0.07)	(-0.04–0.07)	(-0.04–0.06)				
Perceived social exclusion					0.06**	0.01	0.01	0.01
					(0.02–0.11)	(-0.03–0.06)	(-0.04–0.06)	(-0.03–0.06)
Socioeconomic covariates		✓	✓	✓		✓	✓	✓
Lifestyle-related covariates			✓	✓			✓	✓
Health-related covariates				✓				✓
Observations	14,045	13,502	13,339	13,128	14,068	13,524	13,350	13,119
Individuals	6,559	6,363	6,312	6,251	6,568	6,372	6,313	6,243
Pseudo R²	0.0001	0.06	0.06	0.06	0.001	0.06	0.06	0.06

Comments: Unstandardized beta coefficients are shown, with 95% CI in parentheses; *** *p* < 0.001, ** *p* < 0.01, * *p* < 0.05, + *p* < 0.10

Socioeconomic time-varying covariates encompass age, marital status, employment status, and household net equivalent income; lifestyle-related time-varying covariates encompass alcohol intake, smoking status, and frequency of sports activity; health-related time-varying covariates encompass self-rated health, physical functioning, depressive symptoms and number of chronic conditions

Increases in loneliness were significantly associated with reductions in the odds of donation in the unadjusted model and when it was adjusted for socioeconomic covariates. However, the results became insignificant when it was adjusted for lifestyle-related and health-related covariates. In contrast, there was a consistent significant association between increases in social exclusion and reductions in the odds of donation (e.g., in the fully-adjusted model, OR: 0.85, 95% CI: 0.73 to 0.99).

Neither changes in loneliness nor changes in perceived social exclusion were significantly associated with log total amount of donations across nearly all model specifications (see Table 3 for further details). It is worth noting that age, sex, and education did not significantly moderate the association of loneliness and perceived social exclusion with donation behavior outcomes (all *ns*).

## Discussion

Using longitudinal data from wave 5 to 8 of the nationally representative DEAS study, our aim was to investigate the association of loneliness, and perceived social exclusion with donation behavior. Our main findings were as follows: increases in loneliness were significantly associated with lower odds of donation after adjusting for socioeconomic factors. However, this association became insignificant when it was adjusted for lifestyle and health-related covariates. In contrast, a significant association was found between increases in perceived social exclusion and lower odds of donation in all model specifications. Neither changes in loneliness nor perceived social exclusion were significantly associated with changes in log amount of donations. Sociodemographic factors did not moderate the identified associations. Our present longitudinal significantly advances our present knowledge mainly based on cross-sectional observational studies or based on social experiments. For example, Oh and Jung [19] found a cross-sectional association between social exclusion and lower donation intentions based on a small sample of individuals aged 40 years and older. In a broader context, it should also be noted that previous research has also identified associations between prosocial behaviour such as voluntary work and loneliness or isolation [32–35].

We assume that the theory of reciprocity in particular may explain our results [36]. More precisely, individuals experiencing an increase in perceived social exclusion might wonder why they should donate to a society from which they feel more and more excluded. They may feel less like part of a community and may also be somewhat less interested in society. This may make donations as an expression of social solidarity less likely. When individuals experience an increase in perceived social exclusion, they may feel somewhat less bound by social norms that encourage donations or prosocial behavior more generally. Increases in social isolation may also be accompanied by decreases in empathy [37], which may partially drive the lack of donations (see also: [38]). Increasing social exclusion can also lead to social withdrawal. This can be accompanied by limited access to information (e.g., about donation opportunities), which may reduce the willingness to donate. Another explanation is that such increases in social exclusion may potentially also encourage individuals to take on other roles (such as providing support within the community or neighbourhood). Cultural factors may also play a role here since they affect the types of assistance that are sought in a given culture.

In our study, increases in loneliness were only significantly associated with the odds of donation in some model specifications. This may be explained by the fact that individuals experiencing increases in loneliness may partly strive for (re-)building social relationships (see: [37]). This may (partly) compensate for potential negative consequences that could arise from a more critical view of charitable organizations. However, these are speculative explanations that should be further examined in upcoming studies.

Several strengths and limitations are worth noting when interpreting our findings. This is the first observational longitudinal study examining the association of loneliness and perceived social exclusion with donation behavior. Data were used from a nationally representative sample of individuals aged 40 years and over residing in private households. Moreover, the longitudinal data structure was exploited by using FE regressions. Using such regression models also helps to reduce the problem of unobserved heterogeneity. Established tools were used to assess loneliness and perceived social exclusion. Moreover, both the general willingness to donate and the amount of the donation were taken into account. However, both are based on self-reports, which may introduce some bias (e.g., social desirability bias or recall bias). Nevertheless, a recall bias may be rather small given the fact that the recall period was restricted to 12 months.

In conclusion, even after adjusting for various covariates, this longitudinal study revealed a significant and robust association between increases in social exclusion and lower odds of donation. Avoiding increases in perceived social exclusion could encourage the decision to donate, an important prosocial behavior, pending further longitudinal evidence.

Future research could also explore the association of loneliness and social exclusion with donations for specific areas (e.g., for human rights, animal shelters, humanitarian crises, health purposes, research in general, etc.). Moreover, the background of perceived social exclusion (e.g., individuals perceiving themselves as self-excluded vs. those excluded by others) could be linked to donation behavior in upcoming research [19]. We presume that chronic loneliness and exclusion in particular can lead to more severe cognitive distortions. We therefore recommend further research into chronic loneliness and prosocial behaviour.

Research is also needed into other ways of reducing inequalities, such as wealth redistribution or political reforms. Moreover, previous research has also emphasised the importance of cultural context (i.e. individualism vs. collectivism) in charitable donations [39]. Thus, it is questionable to what extent these results can be applied to other countries with other cultural values. We therefore recommend cross-country comparisons in future studies.

## Data Availability

The data used in this study are third-party data. The anonymized data sets of the DEAS (1996, 2002, 2008, 2011, 2014, 2017, 2020, 2020/2021, 2023) are available for secondary analysis. The data has been made available to scientists at universities and research institutes exclusively for scientific purposes. The use of data is subject to written data protection agreements. Microdata of the German Ageing Survey (DEAS) are available free of charge to scientific researchers for non-profitable purposes. The FDZ-DZA provides access and support to scholars interested in using DEAS for their research. However, for reasons of data protection, signing a data distribution contract is required before data can be obtained. For further information on the data distribution contract, please see https://www.dza.de/en/research/fdz/access-to-data/application (accessed on 10 June 2025).
